# The association between admission mean corpuscular volume and preoperative deep venous thrombosis in geriatrics hip fracture: a retrospective study

**DOI:** 10.1186/s12891-023-07147-6

**Published:** 2024-01-08

**Authors:** Shuai-Liang Xu, Kun Li, Wen-Wen Cao, Shao-Hua Chen, Shang-Bo Ren, Bin-Fei Zhang, Yu-Min Zhang

**Affiliations:** 1 Department of Joint Surgery, Honghui Hospital, Xi’an Jiaotong University, No. 555 Youyi East Road, Beilin District, Xi’an, 710054 Shaanxi Province China; 2 Xi’an Medical University, Beilin District, Xi’an, Shaanxi Province China

**Keywords:** Hip fracture, MCV, DVT, Complication

## Abstract

**Objective:**

This study evaluated the association between admission MCV and preoperative deep vein thrombosis (DVT) in geriatric hip fractures.

**Methods:**

Older adult patients with hip fractures were screened between January 2015 and September 2019. The demographic and clinical characteristics of the patients were collected at the largest trauma center in northwest China. MCV was measured at admission and converted into a categorical variable according to the quartile. Multivariate binary logistic regression and generalized additive model were used to identify the linear and nonlinear association between MCV and preoperative DVT. Analyses were performed using EmpowerStats and the R software.

**Results:**

A total of 1840 patients who met the criteria were finally enrolled and divided into four groups according to their MCV levels. The mean MCV was 93.82 ± 6.49 (80.96 to 105.91 fL), and 587 patients (31.9%) were diagnosed with preoperative DVT. When MCV was a continuous variable, the incidence of preoperative DVT increased with mean corpuscular volume. In the fully adjusted model, admission MCV was positively correlated with the incidence of preoperative DVT (OR: 1.03; 95% CI: 1.01–1.05; *P* = 0.0013). After excluding the effect of other factors, each additional 1fL of MCV increased the prevalence of preoperative DVT by 1.03 times as a continuous variable.

**Conclusion:**

MCV was linearly associated with preoperative DVT in geriatric patients with hip fractures and could be considered a predictor of DVT risk. The MCV may contribute to risk assessment and preventing adverse outcomes in the elderly.

**Study registration:**

This study is registered on the website of the Chinese Clinical Trial Registry (ChiCTR: ChiCTR2200057323).

## Introduction

Hip fractures are one of the most common types of fractures in the elderly population [1]. The global number of hip fractures is expected to increase from 1.26 million in 1990 to 4.5 million by 2050 [1]. Meanwhile, deep venous thrombosis (DVT) is the most dangerous complication of hip fracture. Medical studies have generally shown that DVT can lead to pulmonary embolism and even death in patients with fracture trauma [2–4]. In addition, functional recovery in patients with DVT will be delayed, resulting in heavier financial and treatment burdens than those without DVT [5]. Therefore, it is of great significance for clinical work to understand the risk factors and epidemiological characteristics of deep vein thrombosis and prevent the occurrence of deep vein thrombosis.

The incidence of preoperative DVT is higher in elderly patients with hip fractures due to senility, medication, and underlying diseases [6–9]. Thrombosis formation is a complex process that requires the combined action of red blood cells, platelets, and white blood cells, which has been widely recognized. In recent years, more and more basic researches support red blood cell plays an essential role in thrombus formation. The red blood cell indexes in routine blood tests include MCV, MCH, and MCHC, which were used to determine red blood cells' size and hemoglobin content. MCV measures the mean size of red blood cells and is proportional to hematocrit [10]. It has been reported that high MCV was correlated with high mortality in patients with acute myocardial infarction, acute decompensated heart failure, and consecutive patients subjected to percutaneous coronary intervention [11–13]. Zhang et al. found that higher admission MCV was an independent predictor of long-term major adverse cardiovascular events [14]. They believe that the possible reason is that the high MCV value leads to the imbalance between the cytoplasm and the nucleus, hinders the flow of red blood cells with poor flexibility through the microcirculation, and damages the antioxidant properties of the red blood cell membrane. In addition, Weisel et al. pointed out that the increase of MCV and erythrocyte membrane hardness would lead to the change of rheological effect, which promotes the adhesion of platelets to the vascular wall, enhances the interaction between platelets and endothelial cells, and enhances the possibility of thrombosis [15].

Unfortunately, clinical studies on the association between admission MCV and preoperative DVT are limited to our knowledge. In addition, the incidence of DVT increases with age, and the relationship has not been explored in the elderly with hip fractures.

Therefore, we aimed to explore the relationship between MCV at admission and preoperative DVT in elderly with a hip fracture through this retrospective study, which will help surgeons to ensure surgical safety, reduce mortality, help individualized risk assessment, and prevent adverse outcomes.

## Materials and methods

### Study design

In this retrospective cohort study, we recruited older adults who had a hip fracture from 1 Jan 2015 to 30 Sep 2019 at the largest trauma center in Northwest China.

This retrospective study was approved by the Ethics Committee of Xi’an Honghui Hospital (No. 202201009). All patients provided informed consent. All human-related procedures followed the 1964 Declaration of Helsinki and its later amendments. The study has been reported according to the STROCSS 2021 guidelines [16].

### Participants

Demographic and clinical data of the patients were obtained from their original medical records. The inclusion criteria were as follows:

(1) Age ≥ 65 years, (2) X-ray or computed tomography diagnosis of the femoral neck, intertrochanteric, or subtrochanteric fracture, (3) patients who were receiving surgical or conservative treatment in the hospital. Informed consent has been signed and agreed to receive anticoagulant therapy to prevent thrombosis, (4) patients with hip fractures were evaluated by a senior orthopedic surgeon based on physical examination and imaging (including X-ray, CT, or MRI).

Exclusion criteria were as follows:

(1) No preoperative ultrasound result on DVT, (2) no MCV results at admission.

### Hospital treatment

Patients were examined using blood tests to prepare for surgery. Prophylaxis for DVT was initiated at admission. Mechanical thromboprophylaxis (foot intermittent pneumatic compression sleeve, 20 min twice a day) was used to prevent DVT. Low molecular weight heparin was injected subcutaneously for patients without contraindications to prevent DVT.

### Endpoint events

The endpoint event in this study was DVT before operation. We used Doppler ultrasonography to diagnose the DVT. The diagnostic criteria are the presence of a constant intraluminal filling defect. Patients were examined preoperatively. All patients underwent ultrasonography of bilateral lower extremities the day before the scheduled surgery.

### Variables

The variables collected in this study were as follows: age, sex, occupation, history of allergy, injury mechanism, fracture classification, hypertension, diabetes, coronary heart disease (CHD), arrhythmia, hemorrhagic stroke, ischemic stroke, cancer, associated injuries, dementia, chronic obstructive pulmonary disease (COPD), hepatitis, gastritis, age-adjusted Charlson comorbidity index (aCCI), time from injury to admission, admission glomerular filtration rate (GFR), admission D-dimer and MCV.

The dependent variable was preoperative DVT, and the independent variable was the MCV. Other variables were potentially confounding factors.

### Statistics analysis

Continuous variables are reported as mean ± standard deviation (SD) (Gaussian distribution) or median (min, max) (Skewed distribution), and categorical variables are given as frequencies and percentages. We used χ2 (categorical variables), the One-way ANOVA test (normal distribution), or the Kruskal-Whallis H test (skewed distribution) to test for differences among different MCV (quartile). We used a univariate and multivariate binary logistic regression model to test the association between MCV and preoperative DVT with three distinct models by stand linear regression. Model 1 was not adjusted for covariates. Model 2 was a minimally adjusted model with only sex adjusted. Model 3 was a fully adjusted model with meaningful variables in univariate analyses and some bias factors (such as diabetes, hypertension and ischemic stroke) generated by univariate analyses.

To account for the nonlinear relationship between MCV and preoperative DVT, we used a generalized additive model and the smooth curve fitting (penalized spline method) to address nonlinearity. Besides, the two-piecewise binary logistic regression model was also used to explain the nonlinearity further. To test the robustness of our results, we performed a sensitivity analysis. We converted MCV into a categorical variable according to the quartile. We calculated the P for trend to verify the results of MCV as the continuous variable and to examine the possibility of nonlinearity.

All analyses were performed using statistical software packages R. (http://www.R-project.org, R Foundation) and EmpowerStats (http://www. empowerstats.com, X&Y Solutions Inc., Boston, MA, USA). Odds ratios (OR) and 95%CI were calculated. A *P*-value < 0.05 (two-sided) was considered to represent statistical significance.

## Results

### Patient characteristics

**Fig. 1:**
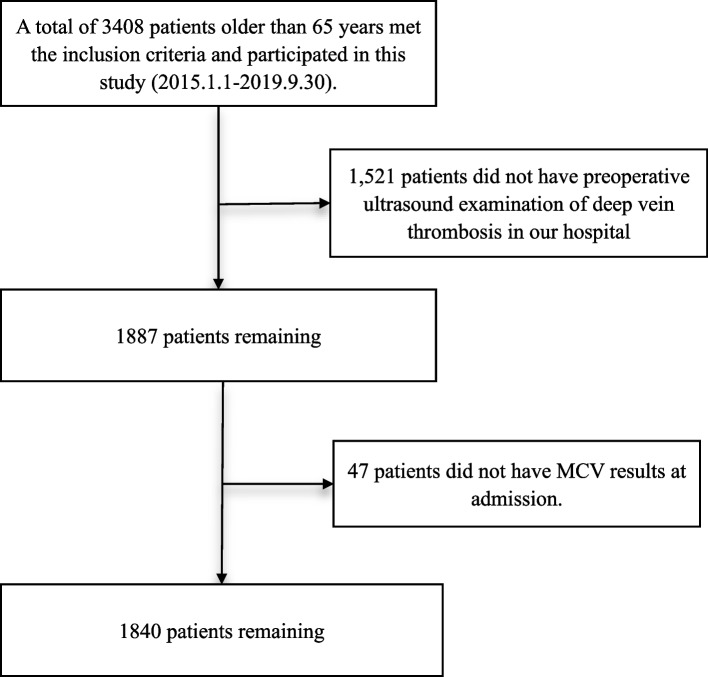
The flow chart of including patient cohort

A total of 1840 patients were included in the study according to the inclusion and exclusion criteria from 1 Jan 2015 to 30 Sep 2019 (Fig. 1). We divided the patients into four groups according to MCV level. The general information of patients is shown in Table 1. Among them, 558 were male, and 1282 were female, with a mean age of 79.40 ± 6.88 (72.52–86.28 years). The injury mechanism included 1773 (96.36%) falls, 53 (2.88%) accidents, and 14 (0.76%) multiple injuries. There were 1106 intertrochanteric fractures, 701 femoral neck fractures, and 33 subtrochanteric fractures. Multiple injuries were in 131 patients. Combined medical diseases: 950 cases of CHD, 924 cases of hypertension, 367 cases of diabetes, 32 cases of hemorrhagic stroke, and 583 cases of arrhythmia. No pulmonary embolism occurred in the preoperative period.

**Table 1:** The Demographic and clinical characteristics according to MCV quartiles

MCV quartiles	Q1	Q2	Q3	Q4	*P*-value	*P*-value^*^
**N**	424	459	471	486		
**MCV**	86.08 ± 5.12	91.82 ± 0.95	95.04 ± 1.08	101.27 ± 4.64	< 0.001	< 0.001
**Age (year)**	79.00 ± 6.93	78.76 ± 6.67	79.03 ± 6.68	80.71 ± 7.06	< 0.001	< 0.001
**Sex**					< 0.001	-
Male	103 (24.29%)	126 (27.45%)	154 (32.70%)	175 (36.01%)		
Female	321 (75.71%)	333 (72.55%)	317 (67.30%)	311 (63.99%)		
**Injury mechanism**					0.182	-
Falling	414 (97.64%)	444 (96.73%)	452 (95.97%)	463 (95.27%)		
Accident	9 (2.12%)	9 (1.96%)	17 (3.61%)	18 (3.70%)		
Other	1 (0.24%)	6 (1.31%)	2 (0.42%)	5 (1.03%)		
**Fracture classification**					< 0.001	-
Intertrochanteric fracture	235 (55.42%)	252 (54.90%)	289 (61.36%)	330 (67.90%)		
Femoral neck fracture	181 (42.69%)	200 (43.57%)	174 (36.94%)	146 (30.04%)		
Subtrochanteric fracture	8 (1.89%)	7 (1.53%)	8 (1.70%)	10 (2.06%)		
**Hypertension**	244 (57.55%)	241 (52.51%)	234 (49.68%)	205 (42.18%)	< 0.001	-
**Diabetes**	126 (29.72%)	103 (22.44%)	83 (17.62%)	55 (11.32%)	< 0.001	-
**CHD**	210 (49.53%)	251 (54.68%)	242 (51.38%)	247 (50.82%)	0.457	-
**Arrhythmia**	128 (30.19%)	139 (30.28%)	148 (31.42%)	168 (34.57%)	0.434	-
**Hemorrhagic stroke**	4 (0.94%)	9 (1.96%)	2 (0.42%)	17 (3.50%)	0.002	-
**Ischemic stroke**	156 (36.79%)	126 (27.45%)	146 (31.00%)	152 (31.28%)	0.029	
**Cancer**	12 (2.83%)	11 (2.40%)	10 (2.12%)	15 (3.09%)	0.792	
**Multiple injuries**	28 (6.60%)	36 (7.84%)	38 (8.07%)	29 (5.97%)	0.542	
**Dementia**	18 (4.25%)	13 (2.83%)	24 (5.10%)	16 (3.29%)	0.28	
**COPD**	21 (4.95%)	26 (5.66%)	30 (6.37%)	29 (5.97%)	0.831	
**Hepatitis**	9 (2.12%)	17 (3.70%)	13 (2.76%)	16 (3.29%)	0.543	
**Gastritis**	3 (0.71%)	7 (1.53%)	11 (2.34%)	6 (1.23%)	0.227	
**DVT**	123 (29.01%)	145 (31.59%)	147 (31.21%)	172 (35.39%)	0.214	
**aCCI**	4.30 ± 1.10	4.14 ± 1.15	4.20 ± 1.13	4.22 ± 1.03	0.171	0.067
**Time to operation (d)**	4.16 ± 2.28	4.27 ± 2.28	4.09 ± 2.34	4.16 ± 2.78	0.748	0.282
**Time to admission (h)**	85.89 ± 217.85	77.78 ± 294.66	92.00 ± 306.33	80.36 ± 179.48	0.836	0.054
**Admission D-dimer**	9.00 ± 19.76	9.39 ± 15.82	9.96 ± 20.98	9.86 ± 16.53	0.855	0.198
**Admission GFR**	78.11 ± 18.12	77.81 ± 17.31	78.07 ± 17.99	75.14 ± 19.28	0.107	0.062

Mean + SD/N (%)*. P*-value*: For continuous variables, we used the Kruskal Wallis rank-sum test and Fisher’s exact probability test for count variables with a theoretical number of < 10

### Univariate analysis of the association between variates and DVT

Based on the results of univariate analysis (Table 2), according to the criteria of *P* < 0.1, we found six confounding factors: sex, fracture classification, multiple injuries, time to operation, dementia, and admission D-dimer.

**Table 2:** Effects of factors on DVT measured by univariate analysis

	**Statistics**	**OR (95%CI)**	***P* value**
**Age (year)**	79.40 ± 6.88	1.00 (0.99, 1.02)	0.7005
**Sex**			
Male	558 (30.33%)	1	
Female	1282 (69.67%)	1.26 (1.01, 1.56)	0.0388
**Injury mechanism**			
Falling	1773 (96.36%)	1	
Accident	53 (2.88%)	1.11 (0.62, 1.98)	0.7206
Other	14 (0.76%)	2.88 (0.99, 8.34)	0.0511
**Fracture classification**			
Intertrochanteric fracture	1106 (60.11%)	1	
Femoral neck fracture	701 (38.10%)	0.68 (0.56, 0.84)	0.0004
Subtrochanteric fracture	33 (1.79%)	1.77 (0.88, 3.54)	0.1069
**Hypertension**	924 (50.22%)	1.13 (0.93, 1.38)	0.2215
**Diabetes**	367 (19.95%)	0.95 (0.74, 1.22)	0.6998
**CHD**	950 (51.63%)	1.07 (0.88, 1.30)	0.488
**Arrhythmia**	583 (31.68%)	1.07 (0.87, 1.32)	0.5182
**Hemorrhagic stroke**	32 (1.74%)	1.68 (0.83, 3.39)	0.1512
**Ischemic stroke**	580 (31.52%)	0.86 (0.69, 1.06)	0.1608
**Cancer**	48 (2.61%)	1.18 (0.65, 2.14)	0.5969
**Multiple injuries**	131 (7.12%)	1.60 (1.12, 2.30)	0.0108
**Dementia**	71 (3.86%)	1.59 (0.98, 2.58)	0.0583
**COPD**	106 (5.76%)	0.88 (0.57, 1.35)	0.5457
**Hepatitis**	55 (2.99%)	0.65 (0.35, 1.23)	0.1849
**Gastritis**	27 (1.47%)	0.48 (0.18, 1.28)	0.1413
**aCCI**	4.21 ± 1.10	0.99 (0.91, 1.09)	0.8784
**MCV**	93.82 ± 6.49	1.02 (1.01, 1.04)	0.0067
**Time to admission (h)**	83.97 ± 255.05	1.00 (1.00, 1.00)	0.2535
**Time to operation (d)**	4.17 ± 2.43	1.05 (1.01, 1.10)	0.0141
**Admission D-dimer**	9.57 ± 18.35	0.99 (0.98, 1.00)	0.0034
**Admission GFR**	77.26 ± 18.23	1.00 (0.99, 1.01)	0.9703

### Multivariate analysis between preoperative MCV and DVT

We used a binary logistic regression model to assess the correlation between admission MCV and preoperative DVT incidence. We showed the non-adjusted model, the minimally-adjusted model, and the fully-adjusted model in Table 3. In the unadjusted model, MCV was associated with preoperative DVT incidence (OR: 1.02; 95% CI: 1.01–1.04; *P* = 0.0067). In the minimally-adjusted model, the results were positively correlated (OR: 1.02; 95% CI: 1.01–1.04; *P* = 0.0041). The results were positively correlated in the fully adjusted model (OR: 1.03; 95% CI: 1.01–1.05; *P* = 0.0013).

**Table 3:** Univariate and multivariate results by linear regression

Exposure	Non-adjusted model	Minimally-adjusted model	Fully-adjusted model
MCV	1.02 (1.01, 1.04) 0.0067	1.02 (1.01, 1.04) 0.0041	1.03 (1.01, 1.05) 0.0013
MCV quartiles			
Q1	1	1	1
Q2	1.13 (0.85, 1.51) 0.4047	1.14 (0.85, 1.52) 0.3751	1.12 (0.83, 1.53) 0.4574
Q3	1.11 (0.83, 1.48) 0.4739	1.13 (0.85, 1.51) 0.3914	1.13 (0.83, 1.54) 0.4256
Q4	1.34 (1.01, 1.77) 0.0405	1.38 (1.04, 1.83) 0.0248	1.47 (1.08, 1.99) 0.0135
*P* for trend	0.0537	0.0321	0.0165

**Data in table**: OR (95% CI) *P*-value

**Outcome variable**: DVT

**Exposed variables**: MCV

**Minimally adjusted model was adjusted for**: Sex

**Fully-adjusted model was adjusted for:** Sex, fracture classification, multiple injuries, dementia, time to operation, admission D-dimer, hypertension, diabetes, ischemic stroke

### Curve fitting and analysis of threshold effect

**Fig. 2:**
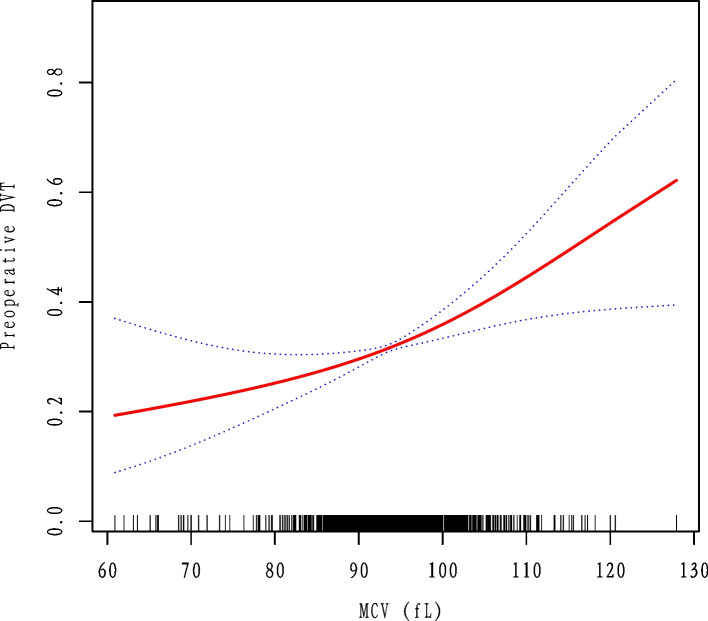
Curve fitting between preoperative MCV and DVT. They were adjusted for sex, fracture classification, multiple injuries, dementia, and time to operation, admission D-dimer, hypertension, diabetes and ischemic stroke

As shown in Fig. 2, there was a linear association between admission MCV and DVT after adjusting for confounding factors. We compared the binary logistic regression model (by stand linear regression) and generalized additive model (two-piecewise linear regression) to explain this association (Table 4), and we observed the inflection point value was 97.9 in the model. However, the *P* for the log-likelihood ratio test was 0.110. It was better to explain the relationship between MCV and DVT by the linear association at present.

**Table 4:** The binary logistic regression model (by stand linear regression) and generalized additive model of preoperative MCV and DVT

Outcome:	OR (95%CI) *P*-value
The binary logistic regression model by stand linear regression	1.03 (1.01, 1.05) 0.0013
The generalized additive model by two-piecewise linear regression	
Inflection point	97.9
< 97.9	1.01 (0.99, 1.04) 0.2208
> 97.9	1.06 (1.02, 1.10) 0.0051
*P* for log-likelihood ratio test	0.110

**Outcome variable:** DVT

**Exposure variables:** MCV

**Adjusted for** Sex, fracture classification, multiple injuries, dementia, time to operation, admission D-dimer, hypertension, diabetes and ischemic stroke

## Discussion

First, this retrospective study found that higher MCV was a risk factor for preoperative DVT. There was a linear association between the admission of MCV and preoperative DVT in geriatric patients with hip fractures. Further, when analyzing as a continuous variable, each additional 1fL of MCV increased the prevalence of preoperative DVT by 1.03 times after excluding the effect of other confounding factors.

As far as we know, Previous studies have shown that the incidence of preoperative lower extremity deep venous thrombosis in elderly patients with hip fracture in China is about 30% [17, 18], similar to our results (31.9%). However, Cho [6] et al. from South Korea reported that 152 elderly patients with a minimum incidence of 2.6% received ultrasound or CT scans, which they attributed to early hospitalization ( 90.1% within three days after injury). This is not easy to achieve in Northwest China. The health resource supply shortage, uneven distribution, and the lack of relevant knowledge of patients lead to delayed admission to hospitals for treatment [19]. Vascular injury caused by fracture activates the coagulation system, and long-term bedridden fixation after fracture can lead to venous congestion and increase the possibility of thrombosis. In addition, the incidence of preoperative DVT in patients with hip fracture is also related to the diagnosis method (including venography, computed tomography, ultrasonography, D-dimer, etc.). Relevant literature points out that the diagnostic rate of different diagnostic methods is from 2.6% to 60% [20]. We used Doppler ultrasound to diagnose deep vein thrombosis, which may impact the incidence of DVT.

This is the first retrospective study on the association between admission MCV and preoperative DVT in geriatrics with hip fractures. Previous studies suggested age, sex, hyperlipidemia, dementia, movement disorder, bedridden time, diabetes, pulmonary disease, d-dimer, and kidney disease as risk factors for developing DVT after fractures [9, 21–24]. Meantime, many studies have found that laboratory indicators of blood routine, such as RDW (red cell distribution width), blood monocyte count, and hematocrit, were risk factors for postoperative deep vein thrombosis in patients with hip fracture [25–27]. There was limited understanding of the risk factors of preoperative DVT after hip fractures, especially the red blood cell indices. Other studies evaluated the relationship between MCV at admission and adverse events in patients with hip fractures and showed varied and conflicting results. The following study by Braekkan SK et al. [28]. found that elevated mean corpuscular volume at admission was not associated with postoperative adverse outcomes in patients with hip fracture. By contrast, Eischer L et al. demonstrated that high hematocrit is a risk factor for Venous thromboembolism (VTE) recurrence in women [27]. Rezende SM et al. found an association between high MCV (above 101.5 fL) and high MCH (above 2.15 fmol) and venous thrombosis [26]. However, for hip fracture patients waiting for surgery, there is limited information on the association between high MCV at admission and preoperative deep vein thrombosis. In this study, we found that MCV was associated with preoperative DVT, and there was a linear relationship between the two factors.

MCV is part of the whole blood count. The calculation method of MCV is the ratio of hematocrit to red blood cell count per liter of blood (Hematocrit is directly proportional to the MCV). They are mainly used for the clinical diagnosis of anemia. According to the relationship between MCV and hematocrit [10] and growing evidence from basic research, we found a potential biological mechanism of high MCV causing DVT. (1) Red blood cells have a typical rheological effect during blood coagulation: The circulation of red blood cells and platelets in blood vessels has specific rules, and red blood cells preferentially move to the center of blood vessels when circulating in blood vessels, resulting in the movement of platelets to endothelial cells (margination), where platelets and blood vessel walls can interact and form a temporary blockage when injured [29–31]. When MCV increases, hematocrit also increases; one consequence of elevated hematocrit is increased margination of platelets, enhancing the interaction between platelets and endothelial cells and promoting thrombosis. (2) MCV is inversely proportional to the deformability of red blood cells. The excellent deformability of red blood cells is mainly the result of their double concave shape, especially the high surface area and volume ratio. Therefore, with the increase of MCV, the degeneration ability of red blood cells decreases, and the rigidity of the red blood cell membrane increases [32, 33], which makes it difficult for red blood cells to squeeze through the microvascular system and strengthens the movement of platelets to the edge, thus promoting the formation of thrombus. The second possibility is that elevated MCV will increase blood viscosity, and viscosity increases exponentially with MCV in large vessels [34], which hinders the speed of blood flow. These hemorheological effects of red blood cells may be a factor that promotes thrombosis because blood flow damage is a component of Virchow’s triad that explains the pathophysiological mechanism of thrombosis through a combination of hypercoagulability, blood flow disorders, and endothelial damage [10].

Furthermore, MCV was affected by many factors, such as age, genetic conditions (such as sickle cell disease and thalassemia), malnutrition (iron, folic acid, or vitamin B12 deficiency), and inflammation [35, 36]. Our study mainly discussed hip fractures in geriatrics. Recently, Hoffmann et al. showed a mean age-related increase in MCV of 6.6% over the entire age range [37], and the MCV was more significant in the elderly. So MCV could be considered a predictor for the risk of DVT.

MCV can be observed in routine blood tests. A blood routine test is a cheap and readily available detection method in most hospitals. Elderly patients with hip fractures after admission were generally required to have a routine blood test, which contains the MCV results. We can use MCV to predict preoperative DVT. MCV can also be used as an indicator in the preoperative DVT prediction model. However, the causal relationship between MCV and DVT needs further exploration. Thus, future research and clinical work should assess the potential role of MCV in preventing and treating thrombosis.

This study has the following notable strengths: First, with a large sample size, we recruited 1840 patients who meet the criteria. To the best of our knowledge, this is the first study of the correlation between admission MCV and preoperative DVT in elderly with hip fractures. Second, we corrected various factors and explored the linear and curvilinear relationships. Through multiple methods, we determine the reliability of the linear relationship. However, the study still has some limitations. (1) We use univariate analysis to select covariates for the model, which is prone to bias. Confounders are associated with both the “independent” and “dependent” variables. We only considered covariates associated with the outcomes. (2) Our data came from one hospital in one region, and there may be selection bias in the study population. Therefore, the extrapolation of the results needed to be confirmed in other populations.

## Conclusion

Taken together, the MCV was associated linearly with preoperative DVT in geriatric patients with hip fractures, and it could be considered a predictor of DVT risk. Further study on the factors that affect the formation of preoperative DVT in geriatrics with hip fractures is conducive to formulating a more appropriate diagnosis and treatment plan.

## Data Availability

Xi’an Honghui Hospital implemented the data. According to relevant regulations, the data could not be shared but could request from the corresponding author.
